# A Comparative Review on Microbiota Manipulation: Lessons From Fish, Plants, Livestock, and Human Research

**DOI:** 10.3389/fnut.2018.00080

**Published:** 2018-09-05

**Authors:** Sylvia Brugman, Wakako Ikeda-Ohtsubo, Saskia Braber, Gert Folkerts, Corné M. J. Pieterse, Peter A. H. M. Bakker

**Affiliations:** ^1^Cell Biology and Immunology Group, Animal Sciences Group, Wageningen University and Research, Wageningen, Netherlands; ^2^Food and Feed Immunology Group, Graduate School of Agricultural Science, Tohoku University, Sendai, Japan; ^3^Division of Pharmacology, Utrecht Institute for Pharmaceutical Sciences, Faculty of Sciences, Utrecht University, Utrecht, Netherlands; ^4^Plant-Microbe Interactions, Department of Biology, Science4Life, Utrecht University, Utrecht, Netherlands

**Keywords:** probiotics, prebiotics, comparative, microbiota, fish, livestock, plants, human

## Abstract

During recent years the impact of microbial communities on the health of their host (being plants, fish, and terrestrial animals including humans) has received increasing attention. The microbiota provides the host with nutrients, induces host immune development and metabolism, and protects the host against invading pathogens (1–6). Through millions of years of co-evolution bacteria and hosts have developed intimate relationships. Microbial colonization shapes the host immune system that in turn can shape the microbial composition (7–9). However, with the large scale use of antibiotics in agriculture and human medicine over the last decades an increase of diseases associated with so-called dysbiosis has emerged. Dysbiosis refers to either a disturbed microbial composition (outgrowth of possible pathogenic species) or a disturbed interaction between bacteria and the host (10). Instead of using more antibiotics to treat dysbiosis there is a need to develop alternative strategies to combat disturbed microbial control. To this end, we can learn from nature itself. For example, the plant root (or “rhizosphere”) microbiome of sugar beet contains several bacterial species that suppress the fungal root pathogen *Rhizoctonia solani*, an economically important fungal pathogen of this crop (11). Likewise, commensal bacteria present on healthy human skin produce antimicrobial molecules that selectively kill skin pathogen *Staphylococcus aureus*. Interestingly, patients with atopic dermatitis (inflammation of the skin) lacked antimicrobial peptide secreting commensal skin bacteria (12). In this review, we will give an overview of microbial manipulation in fish, plants, and terrestrial animals including humans to uncover conserved mechanisms and learn how we might restore microbial balance increasing the resilience of the host species.

## The impact of the microbiota on host health

**Figure 1:**
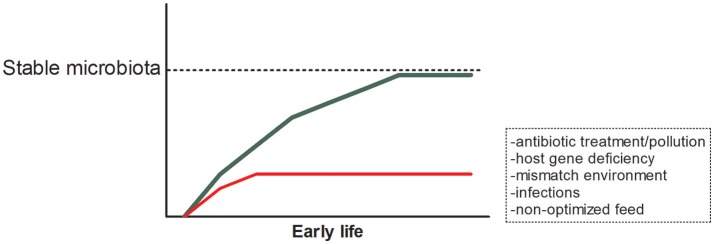
Early life establishment of the microbiota and possible disturbances.

Each organism on earth needs to interact with the vast amount of microbes in its environment. From the moment of exposure both microbes and host interact and react to each other's presence. The current dogma holds that in early life the composition of the microbiota successively leads to the most optimal, healthy, and stable community that strengthens itself and the host. However, when disturbances occur in early life, for example due to antibiotic use, host's inability to interact properly with the microbiota, a mismatch between the environment of parent and offspring, non-optimized feeding conditions or infections, organisms might be more susceptible to disease early and later in life (Figure 1). In this same issue of Frontiers in Microbiology, Ikeda-Ohtsubo et al. describe what is currently known to be the most optimal microbiota in different species and will describe how microbes influence the host in more detail. In this section, we briefly highlight the impact of the microbial composition in relation to host health and continue to discuss manipulation of this composition in subsequent sections. In this review, we decided to focus on less well-reviewed species and did not include studies on rodents. For detailed reviews on (human and) mouse studies we would kindly refer to other reviews (1, 2, 13–15).

### Fish

Since most fish develop from eggs, fish are exposed to the microbial world around them from the moment of hatching. Members of the commensal microbiota present on the outside of the eggs can protect against pathogens, such as Saprolegnia (an oomycete that can infect eggs as well as the skin of fish) (16). Furthermore, colonization of the skin and gills by beneficial bacteria prevents colonization of pathogens through competitive exclusion and their ability to produce antimicrobial compounds specifically targeting certain pathogens (17, 18). An important recent contribution to the field of fish microbiology was made by Sullam et al. They assessed 25 bacterial 16S rRNA data sets from the intestines of several fish species and co-referenced these to free-living and host-associated bacterial communities to understand what factors determine composition (19). In this study Aeromonadales was found to be most abundant in the gut of freshwater species, while Vibrionales dominated salt water species, suggesting that salinity influences microbiota composition. Interestingly, the microbiota of fish herbivores are closely related to those of mammals suggesting that trophic level is of importance. In comparison with free-living bacteria the authors showed that fish gut communities bear resemblance to vertebrate and invertebrate communities strongly suggesting that the intestinal habitat selects those species from the environment that feed of luminal content (specialists).

Upon colonization of the intestines, bacteria increase the renewal rate of the epithelial cell layer lining the gut, and induce a transcriptional program that includes genes involved in nutrient metabolism and immunity (20). Interestingly, several of these induced genes observed in zebrafish upon colonization, are also found when mice are colonized, indicating that the response to bacterial colonization is evolutionary very conserved. Activation of immune cell responses can in turn shape the microbiota. For example, in a small study, it was observed that upon development of adaptive immunity in zebrafish, Vibrionales were specifically repressed by T lymphocytes (8). Research into effects of host genetics on the composition of the microbiota is still in its infancy. However, recent studies performed in zebrafish reveals that host factors can act as an ecological filter, but this can be overwhelmed by other factors, including transmission of microbes among hosts (21, 22).

Several recent studies have made the case that changes in microbial composition (due to antibiotic treatment or changes in feed) might cause disturbance of the microbial community and increase the susceptibility of fish to different pathogens or chemically/feed-induced inflammation (23–26). For example, zebrafish that were pretreated with either colistin sulfate or vancomycine displayed a difference in susceptibility toward chemically-induced intestinal inflammation. Fish that harbored an abundance of Fusobacterial species due to vancomycin pretreatment showed low histological damage to the gut and decreased recruitment of neutrophils to the intestines (23). Piazzon et al. showed that vegetable oil diets fed to gilthead sea bream induced high parasite infection levels decreased growth performance, and decreased intestinal microbiota diversity, while addition of butyrate slightly decreased cumulative mortality after bacterial challenge, did not show the decrease in growth and increased intestinal microbiota diversity (26). Likewise, exposure of catfish to potassium permanganate (PP), a disinfectants used to treat external infections, disturbed the external microbiomes (skin and gills), and increased catfish mortality following experimental challenge with *Flavobacterium columnare* (Columnaris disease) (24). Since, the microbial community of fish appears to be so closely linked to disease resistance and the aquaculture industry is growing rapidly, more research on the influence of microbial populations on fish health is expected to be performed in the near future.

### Plants

The microbiota associated with plants originate from the soil in which the seed germinates and seedlings start to grow (27), but also the seed itself harbors microbes that will colonize the emerging plant (28). Compared to the soil microbiome, the so called rhizosphere microbiome that is associated with the plant roots, contains much higher microbial cell densities and shows higher activity, a phenomenon known as the rhizosphere effect (29). Root exudates create this hotspot for microbial activity (30). The plant microbiome is extremely diverse and the four main associated bacterial phyla are the Actinobacteria, Bacteroidetes, Firmicutes, and Proteobacteria (31). The functional repertoire of plants is greatly extended by the rhizosphere microbiota, including vital functions like increasing nutrient availability, improvement of root architecture, and protection against biotic and abiotic stresses (32). Specific members of the rhizosphere microbiota can protect plants against infectious diseases and mechanisms involved are the production of antimicrobial metabolites and eliciting induced systemic resistance (ISR), in which the plant is primed for enhanced defense (33, 34). The protective effect of the root microbiome against infectious diseases is most obvious in so called disease suppressive soils in which susceptible plants remain healthy despite the presence of a virulent pathogen (35). It was recently demonstrated that the rhizosphere of disease resistant common bean is enriched for plant beneficial bacteria and bacterial biosynthetic genes that encode antifungal traits as compared to disease susceptible bean (36), suggesting that a first line of defense in resistant cultivars is based on the microbiome that they assemble.

### Livestock

#### Pigs

Pork meat is the most consumed meat worldwide with 40,000 tons produced in 2017 (source: OECD, https://data.oecd.org/agroutput/meat-consumption.htm). Raising healthy piglets is of great economic importance. In neonatal piglets before weaning Firmicutes (54%), Bacteroidetes (39%), and Proteobacteria (4%) dominate the fecal microbiota (37). Weaning is a critical period in the piglets life, in which stress and sudden change in diet suppresses their health or can even lead to disease (38, 39). Pigs are generally weaned between 3 and 4 weeks of age (which is much shorter than the natural weaning which occurs around 17 weeks of age). As extensively reviewed by Gresse et al. most studies report a general decrease in diversity as well as a specific decrease in *Lactobacilli* and increase of *Clostridium, Prevotella*, and Proteobacteriacaea around weaning-associated with dietary changes (39). Low levels of antibiotics in feed have been used as growth promotors which also impact the microbiota around this critical weaning period and thereafter (40). Additionally, antibiotic resistance genes derived from phages within the pig microbiota pose a serious problem not only for pig health but also that of humans (41). Post-weaning diarrhea caused by Enterotoxigenic *Escherichia coli* (ETEC) as well as *Salmonella enterica* serovar Typhimurium is a major cause of death of piglets. Outgrowth of these pathogenic species coincides with the reduced diversity of the microbiota observed around weaning possibly enabling pathogens to take up the available niches. Furthermore, increased permeability of the intestines observed around weaning, supplying pathogens with an opportunity to infect (42). Preventing pathogens from colonizing, by providing beneficial bacterial at early age, might improve health of piglets.

#### Ruminants

In ruminants such as cows, a lot of research into microbial manipulation has been geared toward modifying the microbiota to reduce methane emission. Methane emission, partly responsible for climate change, results from digestion of plant-material by methanogenic archaea in the ruminants intestines. Changing the microbiota to increase bacteria that can utilize the methane (such as *Methanobrevibacter* species) might reduce emission (43). The intestinal community of calves changes rapidly after birth and is dynamic during the first 12 weeks of life. *Bacteroides–Prevotella* and *Clostridium coccoides–Eubacterium rectale* species dominate the calves microbiota in this period (44, 45). After weaning, the microbiota changes, and this unstable populations has to cope with a sudden change in diet. As in pigs and other mammals, weaning is considered a critical period, where numerous factors can affect the microbiota as well as health. In calves it was shown that upon weaning Bacteroidetes decreased (still remaining the dominant phyla), while Proteobacteria and Firmicutes increased (46). A change in diet and subsequent change in microbiota has been associated with development of sub-acute ruminal acidosis (SARA) (47). SARA (a reduced ruminal pH; < 5.6 for more than 3 h/day) is the result of dietary shifts, leading to accumulation of volatile fatty acids generated by microbial digestive processes (48, 49).

#### Poultry

The ceca are the gastrointestinal organs that contain the highest microbial density and perform most of the fermentation in chicken. Mainly Firmicutes, Bacteroides, and Proteobacteria (Clostridial species) are found in the ceca of chicken [reviewed in Oakley et al. (50)]. Since eggs are separated from the hens before hatching the specific farm environment in which the eggs hatch supplies the environmental microbes for colonization. Already in the 70s and 80s of the last century it was shown that chicks receiving adult microbiota were resistant toward colonization by *Salmonella* (51). This competitive exclusion concept has helped our understanding of colonization processes of possible pathogenic (pathobionts) species, although fundamental knowledge on the mechanisms are still unclear. Other pathogens in chicken broilers are *Eimeria* and *Clostridium perfringes. Clostridium perfringes* is the causative agent in necrotic enteritis (NE) in poultry. Necrotic enteritis causes tremendous losses in the poultry industry (52). As holds true for most pathobionts like *C. perfringens*, it is clearly involved in the onset of NE, but development of the disease is a multifactorial, multistep process (53). Studies report that co-infection with *Eimeria* increases the chance of NE, since *Eimeria* induce mucogenesis, providing *C. perfringens* with a substrate on which it can grow (54, 55).

### Humans

The human gut microbiota, composed of trillions of individual microbes, is a complex and dynamic system crucial for the development and maturation of both systemic and mucosal immune responses. The early postnatal life is an important period for the colonization of the host microbiota impacting on host health during infancy and even throughout the entire lifespan (56–58). Colonization even starts before birth, possibly via prenatal maternal microbial transmission (59). This initial colonization does not only influence immune development, but also gut maturation, brain, and metabolic development (1).

The development and composition of the infant gut microbiota is shaped by host genetics and different environmental factors, including gestational age, delivery mode (cesarean vs. vaginal delivery), antibiotic use, stress, and diet (breast vs. formula) (60).

Dysbiosis, may drive predisposition to diseases later in life and has been linked to the pathogenesis of several gastrointestinal diseases, like irritable bowel syndrome, inflammatory bowel disease, and celiac disease, indicating that a balanced and diverse microbial community is essential for human health (61). A variety of other inflammatory or immune-mediated diseases, including diabetes, obesity, atopic diseases, and chronic kidney diseases, might largely originate from changes in gut microbiota as well (62, 63).

The microbiota and the immune system are involved in a complex crosstalk and the importance of the elaboration of gut microbiota-generated metabolites and recognition of bacterial epitopes by both intestinal epithelial and mucosal immune cells is clearly described. However, the complete mechanisms by which intestinal microbiota regulates host immunity remain undefined (64, 65).

## Pro- and prebiotics

Probiotics are defined by the World Health Organization's (WHO) to be live micro-organisms that when administered in adequate amounts, confer a health benefit to the host. Prebiotics are ingredients in food such as fibers and oligosaccharides that induce the growth or activity of beneficial microorganisms. In the following section, we will give some highlights of the use of pre- and probiotics in fish, plants, live-stock animals, and humans.

### Fish

#### Prebiotics

Prebiotics are indigestible fibers that are mainly fermented by the microbes in the intestines. The effect of mannan-oligosaccharides (MOS), derived from the cell wall of yeast (*Saccharomyces cerevisiae*) has been studied in different fish species. In common carp fingerlings, feed containing different inclusion levels of MOS (0, 0.05, 0.10, and 0.20%) were evaluated. Total intestinal bacterial counts were not affected, however, there was an increased abundance of lactic acid bacteria levels in fish fed with MOS supplemented feed at the 0.20% inclusion level after 8 weeks of feeding, which might be beneficial to the fish (66). In European sea bass, inclusion of 0.40% of MOS reduced mortality after anally inoculated *V. anguillarum* from 66 to 12.5%, compared to fish fed control diet (67). Likewise, MOS enhanced innate immune responses, led to increased gut mucus production and increased the density of eosinophils in the gut mucosa of European sea bass (68, 69). A study performed in hybrid striped bass revealed that supplementation with MOS changed the microbiota. This study, although small and DGGE based, showed that the dominant species detected in control fish is *Clostridium botulinum* which was not detected in fish fed MOS and other prebiotics, which indicates that prebiotics can reduce the abundance of a specific pathogens (70). Furthermore, 0.2% MOS supplementation in juvenile trout significantly reduced the levels of health threatening *Aeromonas/Vibrio* spp. (from 37 to 9%) (71). For an extensive review of studies on the use of MOS and Galacto-oligosaccharide (GOS) in Gilthead seabream and European seabass we refer to Carbone and Faggio (72).

Artemia (live feed for fish larvae) fed a combination of Fructo-oligosaccharide (FOS) with probiotic *Pediococcus acidilactici* (synbiotics: Pre + Pro), also increased the abundance of lactic acid bacteria in Angelfish after 7 weeks of feeding (73). Likewise, Hoseinifar et al. observed an increase in the heterotrophic aerobic bacteria and lactic acid bacteria in fish fed diets supplemented with 2 and 3% FOS (74). An increase in *Lactobacillus* levels was also observed in stellate sturgeon fed diets containing 1% FOS (75).

Some essential oils from plants might have an impact on the microbiota and host health and might therefore also be termed prebiotic. For example, when juvenile hybrid tilapia were fed 200 mg/kg Next Enhance feed 150 (NE) containing equal levels of thymol and carvacrol (essential oils of oregano) for 6 weeks, the phagocytosis activity of head kidney macrophages was enhanced as well as plasma lysozyme activity (76). Interestingly, when germ free zebrafish were colonized with the microbiota from these NE fed tilapia, they showed attenuated induction of immune response marker genes serum amyloid a, interleukin 1β, and interleukin 8, indicating that these essential oils might change the microbiota and subsequently influence host' immune responses.

#### Probiotics

Although most studies associate the change in microbiota levels (increased *Lactobacillus* abundance) with improved health outcomes, the mechanism by which increases in Lactobacilli ameliorate fish health still needs to be demonstrated. However, most probiotics currently used in aquaculture belong to the lactic acid bacteria group such as *Lactobacillus plantarum, Lactobacillus rhamnosis*, and *Lactobacillus lactis* [reviewed in Banerjee and Ray (77)]. Interestingly, *Phaeobacter inhibens* fed Artemia decrease mortality in sea bass larvae, and the probiotic fed sea bass larvae were more resistant toward *Vibrio harveyi* infection (78, 79). *Phaeobacter* produces Tropodithietic Acid (TDA), which *in vitro* has been shown to inhibit the growth of several pathogenic bacteria, such as *Pseudomonas* and *Aeromonas*. *In vivo, P. inhibens* appears to specifically inhibit *Vibrio* species in the aforementioned sea bass larvae, but also in copepod cultures (78, 79).

In conclusion, pre- and probiotic supplementation of fish feed is a promising alternative for antibiotic treatment in aquaculture. However, a cautionary note on these data are the findings of Cerezuela et al. showing that diets supplemented with probiotic *Bacteroides subtilis* together with prebiotic inulin caused intestinal edema and inflammation in gilthead sea bream (80). This last example, clearly shows the need for in depth studies into the effects of known pro- and prebiotics in the different (aquaculture and model) fish species.

### Plants

#### Prebiotics

The use of organic soil amendments to promote plant growth is common practice in agriculture (81). Apart from adding nutrients that are essential for plant growth, microbiota associated with these amendments and effects of the amendments on the resident microflora are suggested to govern beneficial effects of such additives (82). In a recent study it was demonstrated that addition of broccoli residues or crab meal amendments to soil resulted in a transition of disease conducive to suppressive soil (83). Eggplant is susceptible to wilting caused by the fungus *Verticillium dahliae*, but when grown in soil amended with green manure (broccoli residues) or the chitin containing crab shell meal, it was protected against the disease. In the amended soils bacterial genera with antifungal activity were more abundant and chitinase activity was increased (83). These results show that organic amendments can modify the soil microbiome and support microbiome activities that effectively suppress soil borne disease.

Thermal degradation of organic material by pyrolysis results in the production of biochar, a possible means to sequester carbon and to mitigate climate change (84). Soil amendment with biochar can improve soil fertility and has been reported to influence diseases caused by soil borne plant pathogens. Addition of biochar significantly impacts the soil microbiome and functions (85), suggesting that it may modulate the rhizosphere microbiome of plants grown on this soil and thereby affect disease incidence and severity. Indeed biochar amendments have been shown to reduce disease, but if this is due to the ISR eliciting activity of biochar itself or if it results from modulation of the microbiome remains to be elucidated (86).

Thus, addition of prebiotics to control plant diseases follows as yet a trial and error approach. Discovering new prebiotics may result from studies in which effects of specific plant produced compounds on beneficial microbes are recorded. Recently it was suggested that scopoletin, a compound excreted by Arabidopsis roots upon colonization by beneficial bacteria, can support the beneficial bacteria (87). Application of such plant derived prebiotics may lead to stimulation of beneficial microbes within the resident microbiota or may be used to sustain populations and activities of introduced biocontrol agents.

#### Probiotics

A wealth of literature is available of studies on application of so called biological control agents that can benefit plant growth by suppressing diseases, and their modes of action have been studied in detail (88, 89). The best studied bacterial biological control agents are *Bacillus* and *Pseudomonas*. When applied to seed or as a soil drench, specific strains of *Bacillus* and *Pseudomonas* spp. can protect plants against soil borne pathogens (33, 90–92) Effects of these biological control agents can be based on direct inhibition of the pathogen through the production of antimicrobial compounds, eliciting ISR, or a combination of both modes of action (33, 34). The ability of the bacteria to colonize the plant root, also referred to as rhizosphere competence, is crucial for their biocontrol efficacy (93). Inconsistency in the performance of biological control agents is often related to poor establishment of the bacteria in the rhizosphere, resulting in population densities that are below the threshold levels needed for effective biocontrol. As a result many potential biological control agents have been identified over the last decades but relatively few have been developed into commercial products. Commercialized bacterial biocontrol agents include well-studied *Bacillus* and *Pseudomonas* spp. (94). In view of the urgent need of alternatives for chemical control of plant diseases companies have great interest in developing new and innovative commercial products. Recent insights from metagenomic studies suggest that microbial consortia are involved in soil suppressiveness (11, 95, 96), and thus mixed inocula are more likely to be effective in plant protection than single inocula. Moreover, it has been hypothesized that domestication of our crop plants has resulted in degradation of their root microbiome composition and function (5, 97) and thus breeding programs that also focus on a healthy microbiome are crucial to further develop sustainable crop production.

### Livestock

#### Pigs

##### Prebiotics

Especially with realization that use of in-feed antibiotics as growth promotors can greatly disturb the microbiota in post-wean piglets, studies are aimed at providing prebiotics that can restore beneficial microbes in the gut of piglets. As with most species studied, prebiotics used in pigs are GOS, FOS, MOS, and Cello-oligosaccharides (COS). It was shown that COS increased lactobacilli in jejunal and colonic contents (98). Furthermore, COS increased epithelial barrier function shown by decreased leakage of fluorescein isothiocyanate (FITC-) dextran 4 kDa in jejunum and colon and increased trans-epithelial electrical resistance (TEER) in Ussing chamber experiments. In line with this several tight junction proteins were increased (98).

Alizadeh et al. investigated the effect of GOS in early life. Piglets received a milk replacer with or without the addition of GOS for 3 or 26 days. Dietary GOS increased *Lactobacilli* and *Bifidobacteria* numbers at day 26. Addition of GOS to the diet of piglets increased defensin porcine β-defensin-2 in the colon and secretory IgA levels in saliva (99). In contrast, in another study were neonatal piglets were given formula supplemented with GOS and polydextrose (PDX) *Lactobacillus* spp. were not increased (100). In all of these studies age of the piglets and duration of the supplementation is different, therefore it is difficult to compare the outcomes. Interestingly, a recent study evaluated the effects of maternal prebiotic consumption on offspring intestinal defenses and immune system responsiveness. The authors showed that maternal short chain FOS supplementation improved ileal cytokine secretions and increased IgA vaccine response to *Lawsonia intracellularis* in the serum and ileal mucosa (101).

In a study assessing the synbiotic effect of FOS and *Bifidobacterium animalis*, Trevisi et al. observed in 21–35 day old piglets that *B. animalis* fed together with FOS (2%) increased TLR2 expression in the lymph node, but did not reduce bacterial translocation (102).

##### Probiotics

In order to reverse deleterious effects of weaning on microbiota stability there have been numerous studies performed using probiotics in post-weaning piglets. The most frequently used probiotics are members of *Lactobacillus, Bifidobacterium, Enterococcus*, or *Streptococcus*. However, studies are difficult to compare and results may differ from farm to farm. For a comprehensive review on probiotic use and its challenges in pigs we refer to Barba-Vidal et al. that critically reviews the most recent literature on this topic (103).

#### Ruminants

##### Prebiotics

Similar to pigs, in calves several types of oligosaccharides have been tested for their health increasing or disease preventing activities (by prevention of pathogen binding to epithelial cells). MOS addition to the diet of 5 day old calves (4 g/calf/day up to 2 months of age) improved growth and decreased the number of coliforms in feces (104). GOS added to the diet of calves promoted *Lactobacillus* and *Bifidobacteria*, but due to its laxative effects had lower growth performance (105). Addition of short chain fructo-oligosaccharides (scFOS) to the diet of 8–10 day old calves increased butyrate production while reducing acetate production, which might have health effects, although only growth was assessed in this study (106). Few studies assessed immunological parameters to investigate whether prebiotics convey health effects. Fleige et al. assessed whether long-term lactulose feeding combined with *Enterococcus faecium* affected immune cell activation markers, cytokine responses and IgA Fc-receptor (107). Supplementation of calf feed with 3% lactulose increased the number of blood lymphocytes. Also a small increase in the expression of IgA Fc-receptor was observed in the ileal mucosa in male calves receiving 1% lactulose, but this was not significantly different in the 3% group. Furthermore, the authors report effects on CD4+ (lower in ileum in lactulose group) and CD8+ (higher in blood of females) T cells as well as decreased levels of IL10 and Interferon gamma in the ileum. Feeding calves COS increased the proportion of *C. coccoides–E. rectale* group, while it had no effect on *Bifidobacteria* and *Lactobacilli*, but did increase butyrate levels, which could have beneficial effects (108, 109).

##### Probiotics

To counteract low ruminal pH that might cause SARA, species that keep lactate levels stable such as *Enterococcus* and *Lactobacillus*, or that feed on lactate (Megasphaella or Propionibacteria) are used (110–113). In addition yeast is given to aid the digestion of cellulose. Besides studies that show a beneficial effects of pre- or probiotics on the health of calves, there is an equal amount not showing effects [summarized in Uyeno et al. (114)]. This discrepancy in the data might result from farm to farm differences and the health status of the calves at baseline.

#### Poultry

##### Prebiotics and probiotics

For an overview on the effects of prebiotic supplementation on the microbiota and health of chicken we refer to an excellent recent review by Pourabedin and Zhao (115). In summary, research has been done on the prebiotic effects of MOS, FOS, XOS (xylooligosaccharides; degradation products of lignocellulose materials), GOS, SMO (soybean meal oligosacchahides), and mainly found, or investigated effects on *Lactobacillus* and *Bifidobacteria*. Future research will focus more on metabolites such as short chain fatty acids (SCFAs) and other possible beneficial microbes that can be discovered as more in depth sequencing of chicken microbiota is undertaken.

### Humans

#### Prebiotics

There is growing recognition of the role of nutritional and therapeutic strategies, including pro- and prebiotics, in targeting the composition and the metabolic activity of the gut microbiota, which can in turn impact human health.

Human milk oligosaccharides (HMO) are the first prebiotics in humans that are essential for postnatal growth and development of the gastrointestinal and immune system as demonstrated by comparing breast-fed infants with formula-fed infants (116). HMO facilitate the establishment of the microbiota, stimulate intestinal development, promote intestinal development and prevent pathogenic infections, as reviewed by Donovan and Comstock (117). Alternatives for HMO, including GOS and FOS, are used in infant formula. These non-digestible oligosaccharides (NDO) have several beneficial health effects. NDO are known to reduce the incidence of allergic manifestations (118–120), stimulate the vaccine-specific immune response (121) and protect against infections (122, 123). GOS are also effective in alleviating symptoms of chronic inflammatory diseases, like irritable bowel syndrome (124) and reduce the prevalence of diarrhea (125), while FOS can promote satiety and weight loss in obese patients (126). There is also evidence that prebiotics can impact various biomarkers of colorectal cancer (127, 128) and Abrams et al., showed that inulin-type fructans enhances mineral absorption, including calcium, and bone mineralization [Abrams et al. (129), also reviewed by Slavin (130)].

These health effects were related to their prebiotic effect, including changes in microbiota composition and stimulation of growth and activity of health-promoting *Lactobacilli* and *Bifidobacteria* (131–134), but can also be caused by fermentation products of these bacteria, such as SCFAs (135–137). Moreover, oligosaccharides inhibit the adhesion of pathogens on the epithelial surface (138–140).

The impact of prebiotics on the microbiota influence immune signaling as shown by beneficial effects on the mucosal immune system and gut-associated lymphoid tissue (GALT), increased secretory IgA and mucosal Ig, increasing anti-inflammatory cytokines, decreasing pro-inflammatory cytokines and altering lymphocyte expression [reviewed by Shokryazdan et al. (141) and Wilson and Whelan (142)]. However, there are also microbiota-independent effects of NDOs. Epithelial cells and immune cells can directly interact with oligosaccharides to modulate an immune response, for example via activation of peptidoglycan recognition protein 3 (PGlyRP3) and peroxisome proliferator-activated receptor γ (PPARγ), carbohydrate receptors, such as C-type lectin or Toll-like receptors (TLRs), including TLR4, nucleotide oligomerization domain containing proteins (NODs), 2 and via galectins (143–145). Although, one should take care that, when investigating receptor-mediated signaling of oligosaccharides that the preparations are devoid of LPS contamination, since already small amounts can have effects on immune cells (146). In addition, prebiotics have stabilizing effects on the intestinal barrier and protect against barrier impairment (147–150). The structure, chain length, solubility, fermentability, and viscosity of oligosaccharides are important characteristics that possibly determine the health effect in the host (150–152). Among different dietary compounds, omega-3 (n-3) polyunsaturated fatty acids (PUFA) in the diet have demonstrated a beneficial impact on the intestinal microbiota composition and development (153–155). Polyphenols (e.g., flavonols and quercetin) are a hot topic for future nutritional strategies related to their biological activities, including antimicrobial, antioxidant, or anticarcinogenic activities, and modulation of the gut microbiota by stimulation of beneficial bacteria (156–158). Besides polyphenols, other minor food compounds, including zinc, conjugated linoleic acid, L-carnitine, choline, sphingomyelin, or ellagitannins have been reviewed by Roca-Saavedra et al. (159) to modify the intestinal microbiota and consequently, impact human health.

#### Probiotics

The complex microbial communities that colonize the human gastrointestinal tract are important in human health and modulation of the intestinal microbiota composition is one of the potential health-beneficial effects of probiotics (160). Probiotics are not only used to maintain and stimulate a healthy microbiota in healthy individuals, but there is increasing scientific evidence that probiotics can be used for prevention and treatment of a large number of disease states and intestinal disorders associated with an unbalanced intestinal microbiota (dysbiosis). The efficacy of probiotics have been demonstrated in diarrhea induced by antibiotics or infections, neonatal necrotizing enterocolitis, inflammatory bowel disease, and irritable bowel syndrome, *Helicobacter pylori* infection, lactose intolerance, and metabolic syndromes (161–163). Various meta-analyses and systematic reviews indicate encouraging effects of probiotics on allergy, atopic diseases, and respiratory infections (164, 165). There is strong evidence that there are interactions between the gut microbiota and the nervous system, therefore the use of pre- and probiotics in preventing or treating neurologic diseases is a topic of great interest (166, 167).

Since clinical benefits of probiotics depend on strain selection, delivery method, dosage, and duration of administration, as well as their ability to survive the stomach pH and reach the the GI tract (168) discrepancies between studies are observed.

*Bifidobacterium, Lactobacillus*, and *Saccharomyces* are well-known probiotics widely used and studied for improving human health. Probiotics do not always colonize the intestinal tract to exert their benefits, but can also remodel or influence the existing microbiota. Plausible mechanisms, by which probiotics are able to modify the intestinal microbiota and/or induce responses potentially beneficial to the host are: competition for nutrients (and prebiotics), reduction of the luminal pH, induction, and secretion of antimicrobial factors (e.g., bacteriocins, defensins), SCFA production, prevention of pathogen adhesion to epithelial cells, improvement of intestinal barrier function (e.g., via decreased apoptosis of epithelial cells, increased mucin production, and/or modulation of tight junction proteins), modulation of immune responses (e.g., via increasing mucosal immunity, regulating Thelper cell responses, and release of cytokines) (160, 162, 169, 170). Probiotics and/or their soluble factors can communicate with intestinal epithelial cells via TLRs, and in addition, can be transported across the intestinal epithelium by M cells and may elicit the immunomodulatory effects by activating the APCs, influencing the systemic immune responses (171).

## Microbiota transfer

### Fish

Studies performed in zebrafish and mice revealed that microbial communities are assembled in predictable ways. Rawls et al. transplanted mouse intestinal content into zebrafish and vice versa to investigate whether microbial communities are shaped by the host. It was found that the transplanted community resembled its community of origin (donor) in terms of the species that were present, but the relative abundance of these species changed to resemble the normal gut microbial community composition of the recipient host. This means that microbial communities arise in part from distinct selective pressures imposed within the gut habitat of each host (7). Recent studies using large numbers of zebrafish showed that host factors can act as an ecological filter, but this can be overwhelmed by other factors, including transmission of microbes among hosts (21, 22). This suggests that microbial management in fish must be targeted at group level and that microbial transplants in fish might be a little more challenging.

### Plants

A classic experiment in studies on disease suppressive soils is the transfer of tiny amounts of suppressive soil to a disease conducive soil, leading to the transfer of disease suppressiveness. One of the first documented transfer experiments was published in 1931 by Henry. In this study an amount of microbially active soil that adhered to the tip of a moist sterile platinum needle was transferred to 50 g of sterilized soil and resulted in almost complete suppression of *Helminthosporium* foot rot of wheat. Such transfer experiments have since then been used to study the involvement of microbes in disease suppressive soils. In disease suppressive fields plants are protected against disease, whereas in adjacent fields with similar chemical and physical soil conditions plants become diseased. The best studied example is take-all decline of wheat, in which soil that is cultivated to wheat continuously develops suppressiveness against the take-all pathogen *Gaeumannomyces graminis* var. *tritici* (35). Upon mixing take-all suppressive into conducive soil in a 1:9 ratio, the resulting mix was suppressive to the disease (172). Similar experiments have been reported for *Rhizoctonia solani* suppressive sugar beet fields (11) and *Fusarium oxysporum* suppressive strawberry fields (173). Using both cultivation dependent and sequencing based cultivation independent methods, microbes and functions involved in the control of the disease were identified in the above mentioned studies. Obviously transferring 10% suppressive soil into a conducive soil is unrealistic in practice, but elucidating the mechanisms underlying suppressiveness and especially deciphering how plants assemble their disease suppressive microbiomes will be instrumental in sustaining healthy plant microbiomes. For many suppressive soils a severe disease outbreak is needed for suppressiveness to develop, suggesting that both the plant and the pathogen need to be present to assemble a protective microbiome. Thus, it was postulated that plants can “cry for help” upon pathogen attack resulting in specific changes in their microbiomes. In *Arabidopsis thaliana* it was demonstrated that aboveground infection with the downy mildew pathogen *Hyaloperonospora arabidopsidis* results in the assemblage of a plant beneficial bacterial consortium, and effects of the disease induced changes can protect a new population of plants growing in the same soil (96). Uncovering plant cues that govern this disease induced microbiome assemblage will facilitate directed manipulation of the rhizosphere microbiome in a sensible manner.

### Livestock

In ruminants, transfaunation of ruminal contents, which contains protozoa, bacteria, and methanogenic archaea as major components, has been a common treatment to improve rumen functions and milk production (174). Studies have shown that the ciliated protozoa responsible for digestion of plant materials, can be successfully transferred, but bacterial community seems to be more resistant, which may be due to high host-specific properties (175). While an early study has shown that inoculation of fecal microbiota of healthy adult broilers could reduce the number of *Salmonella infantis* in newly hatched chickens (51), fecal microbiota transplantation (FMT) from chickens with good feed efficiency has not been proved to be effective for modulating the feed efficiently of recipient chickens (176). Similarly in pigs, both positive and negative effects of FMT have been reported. Weight gain and improved innate immunity as well as low rate of diarrhea of piglets orally inoculated with fecal microbiota suspension of healthy adult have been reported (177). In contrast, McCormack et al., have reported that FMT from highly feed-efficient pigs have not been able to deliver the donor phenotype but rather had detrimental effects on recipient sows and their piglets, while increased innate host defense signaling has also been observed, which can be attributed to the altered intestinal microbiota as has been shown in the Hu's study (178). Ribeiro et al. have made an interesting attempt to transfer rumen contents of bison, which may be efficient at digesting low-quality forages, to cattle and found increased protein digestibility and nitrogen retention, while fiber digestibility was not improved (179). This kind of wild-to-domestic microbiota transplant would be an intriguing strategy to regain “extinct” microbial members through domestication, but microbial transfer between genetically different animals should be carefully conducted, since the treatment could be resulted in disturbance of original microbiota (180). In general, the major obstacle of FMT in young livestock animals for meat production may be that FMT can interrupt normal microbial acquisition such as parental transfer. Therefore, understanding the developmental timeline of intestinal microbial community assemblage of the targeted animals would be crucial for contriving future FMT strategies (181).

### Humans

In order to restore a healthy balance between human host and microbes, there has been growing interest in the use of FMT, which entails stool transfer from a healthy donor into a patient's intestine. This technique durably alters the gut microbiota of the recipient. The introduced bacterial strains are easily accepted and persist in an established microbial community in the intestine, however, individual differences of microbiota resistance and donor-recipient compatibilities are indicated after FMT (182).

The microbial community for transplantation can be instilled by various methods, including nasogastric or nasointestinal tubes, endoscopy, colonoscopy, rectal tubes, sigmoidoscopy, enema, or encapsulated formulations, or a combined approach, but there is no clear consensus regarding the optimal instillation method (183). To date, most clinical experience has focused on the use of fecal transplants in patients with recurrent *Clostridium difficile* infection and FMT has become established as a highly efficacious and safe treatment method for these patients (183, 184). It is actively studied as treatment option in inflammatory bowel disease, irritable bowel syndrome and metabolic syndromes, however, evidence is still limited and more randomized controlled trials are needed (184–187). FMT may also have potential applications in a variety of other conditions associated with intestinal dysbiosis, including neuropsychiatric disorders, allergic disorders, and auto-immune disorders as reviewed by Xu et al. (188).

Future work will focus on the standardization of donor screening/selection, feces preparation, clinical application, microbiome analysis, obtaining more robust (long-term) safety data, excluding unwanted co-transfer of pathogenic microbes, the understanding of the exact microbial recovery mechanism (183, 186, 189, 190). Especially, determining what constitutes a healthy microbiota that can be safely transferred will still needs more fundamental research.

## Conclusion

**Figure 2:**
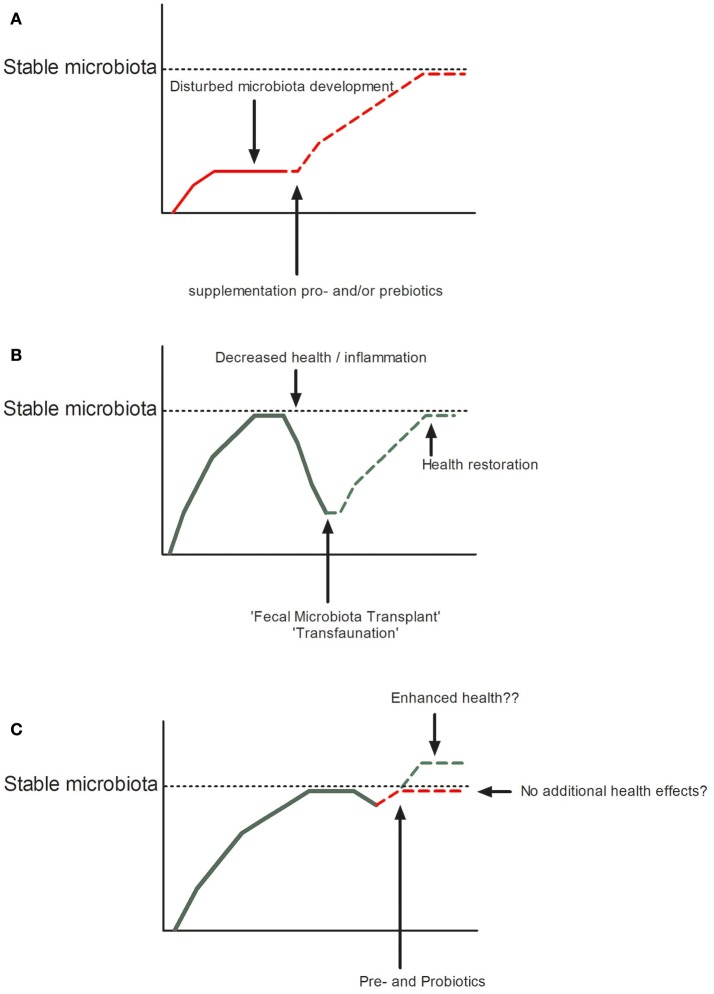
Restoration or improvement of microbial homeostasis in disturbed (**A**: by pro- or prebiotics or **B**: Fecal Microbiota Transplant) or undisturbed states **(C)**.

Antibiotic resistance is on the rise, due to many years of large-scale use of antibiotics as growth enhancers in livestock and aquaculture. This poses a threat not only to the health of our production animals but to the human population as well. Legislation preventing overuse of antibiotics has led to the rapid emergence of studies into the use of pre- and probiotics in fish, plants, livestock animals, and humans. As we have tried to illustrate in this review, a lot of progress has been made. Pre- and probiotics are used to increase early life health and help reach a stable healthy microbiota and fecal transplants have been shown to successfully restore health (Figure 2). Whether pre- and probiotics can enhance health when a stable microbiota is in place still remains an open question. Although from the above mentioned studies it has emerged that oligosaccharides may stimulate certain beneficial microbes to persist or even become dominant, most of the time we do not understand the mechanism by which these species influence the health of their host, if at all. Dosing, duration, and age at which pre- and probiotics are given might all determine whether one observes effects or not. As for the effect of probiotics, are these effects transient, or do *Lactobacilli* and *Bifidobacteria* need to persist for a long time to elicit their health effects. Furthermore, what determines whether a microbe is beneficial within the genera of *Lactobacilli* and *Bifidobacteria*? This might as well be host-specific. Considering the interplay between the host and members of the microbiota selecting a “healthy microbiota” for fecal transplantation may not be as straightforward as we now believe. Depending on the genetic make-up of the individual, microbes might behave different in different hosts. Specifically, we need to make sure we are not copying that what works in one species directly to other species without understanding the effects. In fish for example, the ratio Bacteroidetes/Proteobacteria increases upon inflammation (191), while in humans and mice a decrease of this ratio is associated with inflammation (mainly due to an increase of gamma-Proteobacteria) (192, 193) and in plants Pseudomonas (a gamma-Proteobacterium) is considered to be beneficial (94). Furthermore, some experiments are performed *in vitro* in which the tissue context and immune system of the entire organism is lacking, while others are performed *in vivo* in which one is limited by the read-out parameters that can be investigated or controlled. However, the fact that we know that all hosts select certain species that can protect them from colonization or infection with pathogens is a strong lesson from nature we can exploit in our artificial rearing and culturing conditions in agriculture and human medicine. Furthermore, in an exciting new paper published in Science in 2018 Manfredo Vieira et al. (194) showed that translocation of the gut pathobiont, *Enterococcus gallinarum*, to the liver and other systemic tissues triggers autoimmune responses in a genetic background predisposing to autoimmunity in mice. This illustrates nicely, that we are still in the beginning of understanding host-microbe interaction. Therefore, more fundamental species specific research is needed to fully understand the vast network of interactions between the microbial world and their hosts. Dissecting the intimate relationship between the host and its microbial community can uncover novel mechanisms that might be exploited to restore microbial community structure in those plants and animals that suffer from dysbiosis.

## Author contributions

SyB, WI-O, SaB, GF, CP, and PB all wrote the manuscript according to their expertise and added to different sections. SyB wrote the paragraphs on fish and livestock, WI-O wrote the FMT of livestock, SaB and GF wrote the paragraphs on humans, CP and PB wrote the paragraphs on plants.

### Conflict of interest statement

The authors declare that the research was conducted in the absence of any commercial or financial relationships that could be construed as a potential conflict of interest.

## References

[1] Backhed F Ley RE Sonnenburg JL Peterson DA Gordon JI. Host-bacterial mutualism in the human intestine. Science (2005) 307:1915–20. 10.1126/science.1104816 15790844

[2] Garrett WS Gordon JI Glimcher LH. Homeostasis and inflammation in the intestine. Cell (2010) 140:859–70. 10.1016/j.cell.2010.01.023 20303876 PMC2845719

[3] Dimitroglou A Merrifield DL Carnevali O Picchietti S Avella M Daniels C. Microbial manipulations to improve fish health and production–a Mediterranean perspective. Fish Shellfish Immunol. (2011) 30:1–16. 10.1016/j.fsi.2010.08.009 20801223

[4] Berendsen RL Pieterse CM Bakker PA. The rhizosphere microbiome and plant health. Trends Plant Sci. (2012) 17:478–86. 10.1016/j.tplants.2012.04.001 22564542

[5] Pieterse CMJ de Jonge R Berendsen RL. The soil-borne supremacy. Trends Plant Sci. (2016) 21:171–3. 10.1016/j.tplants.2016.01.018 26853594

[6] Andreote FD Pereira ESMC. Microbial communities associated with plants: learning from nature to apply it in agriculture. Curr Opin Microbiol. (2017) 37:29–34. 10.1016/j.mib.2017.03.011 28437663

[7] Rawls JF Mahowald MA Ley RE Gordon JI. Reciprocal gut microbiota transplants from zebrafish and mice to germ-free recipients reveal host habitat selection. Cell (2006) 127:423–33. 10.1016/j.cell.2006.08.043 17055441 PMC4839475

[8] Brugman S Schneeberger K Witte M Klein MR van den Bogert B Boekhorst J. T lymphocytes control microbial composition by regulating the abundance of Vibrio in the zebrafish gut. Gut Microbes (2014) 5:737–47. 10.4161/19490976.2014.972228 25536157 PMC4615293

[9] Raaijmakers JM Mazzola M. Soil immune responses. Science (2016) 352:1392–3. 10.1126/science.aaf3252 27313024

[10] Sommer F Anderson JM Bharti R Raes J Rosenstiel P. The resilience of the intestinal microbiota influences health and disease. Nat Rev Microbiol. (2017) 15:630–8. 10.1038/nrmicro.2017.58 28626231

[11] Mendes R Kruijt M de Bruijn I Dekkers E van der Voort M Schneider JH. Deciphering the rhizosphere microbiome for disease-suppressive bacteria. Science (2011) 332:1097–100. 10.1126/science.1203980 21551032

[12] Nakatsuji T Chen TH Narala S Chun KA Two AM Yun T. Antimicrobials from human skin commensal bacteria protect against *Staphylococcus aureus* and are deficient in atopic dermatitis. Sci Transl Med. (2017) 9:eaah4680. 10.1126/scitranslmed.aah4680 28228596 PMC5600545

[13] Fiebiger U Bereswill S Heimesaat MM. Dissecting the interplay between intestinal microbiota and host immunity in health and disease: lessons learned from germfree and gnotobiotic animal models. Eur J Microbiol Immunol. (2016) 6:253–71. 10.1556/1886.2016.00036 27980855 PMC5146645

[14] Littman DR Rudensky AY. Th17 and regulatory T cells in mediating and restraining inflammation. Cell (2010) 140:845–58. 10.1016/j.cell.2010.02.021 20303875

[15] Kau AL Ahern PP Griffin NW Goodman AL Gordon JI. Human nutrition, the gut microbiome and the immune system. Nature (2011) 474:327–36. 10.1038/nature10213 21677749 PMC3298082

[16] Liu Y de Bruijn I Jack AL Drynan K van den Berg AH Thoen E. Deciphering microbial landscapes of fish eggs to mitigate emerging diseases. ISME J. (2014) 8:2002–14. 10.1038/ismej.2014.44 24671087 PMC4184010

[17] Carbajal-Gonzalez MT Fregeneda-Grandes JM Suarez-Ramos S Rodriguez Cadenas F Aller-Gancedo JM. Bacterial skin flora variation and *in vitro* inhibitory activity against Saprolegnia parasitica in brown and rainbow trout. Dis Aquat Org. (2011) 96:125–35. 10.3354/dao02391 22013752

[18] Boutin S Bernatchez L Audet C Derome N. Antagonistic effect of indigenous skin bacteria of brook charr (*Salvelinus fontinalis*) against *Flavobacterium columnare* and F. psychrophilum. Vet Microbiol. (2012) 155:355–61. 10.1016/j.vetmic.2011.09.002 21958747

[19] Sullam KE Essinger SD Lozupone CA O'Connor MP Rosen GL Knight R. Environmental and ecological factors that shape the gut bacterial communities of fish: a meta-analysis. Mol Ecol. (2012) 21:3363–78. 10.1111/j.1365-294X.2012.05552.x 22486918 PMC3882143

[20] Rawls JF Samuel BS Gordon JI. Gnotobiotic zebrafish reveal evolutionarily conserved responses to the gut microbiota. Proc Natl Acad Sci USA. (2004) 101:4596–601. 10.1073/pnas.0400706101 15070763 PMC384792

[21] Burns AR Miller E Agarwal M Rolig AS Milligan-Myhre K Seredick S. Interhost dispersal alters microbiome assembly and can overwhelm host innate immunity in an experimental zebrafish model. Proc Natl Acad Sci USA. (2017) 114:11181–6. 10.1073/pnas.1702511114 28973938 PMC5651736

[22] Stagaman K Burns AR Guillemin K Bohannan BJ. The role of adaptive immunity as an ecological filter on the gut microbiota in zebrafish. ISME J. (2017) 11:1630–9. 10.1038/ismej.2017.28 28304369 PMC5520148

[23] Brugman S Liu KY Lindenbergh-Kortleve D Samsom JN Furuta GT Renshaw SA. Oxazolone-induced enterocolitis in zebrafish depends on the composition of the intestinal microbiota. Gastroenterology (2009) 137:1757.e1–67.e1. 10.1053/j.gastro.2009.07.069 19698716

[24] Mohammed HH Arias CR. Potassium permanganate elicits a shift of the external fish microbiome and increases host susceptibility to columnaris disease. Vet Res. (2015) 46:82. 10.1186/s13567-015-0215-y 26170019 PMC4501127

[25] He S Wang Q Li S Ran C Guo X Zhang Z. Antibiotic growth promoter olaquindox increases pathogen susceptibility in fish by inducing gut microbiota dysbiosis. Sci China Life Sci. (2017) 60:1260–70. 10.1007/s11427-016-9072-6 28674769

[26] Piazzon MC Calduch-Giner JA Fouz B Estensoro I Simo-Mirabet P Puyalto M. Under control: how a dietary additive can restore the gut microbiome and proteomic profile, and improve disease resilience in a marine teleostean fish fed vegetable diets. Microbiome (2017) 5:164. 10.1186/s40168-017-0390-3 29282153 PMC5745981

[27] Berg G Smalla K. Plant species and soil type cooperatively shape the structure and function of microbial communities in the rhizosphere. FEMS Microbiol Ecol. (2009) 68:1–13. 10.1111/j.1574-6941.2009.00654.x 19243436

[28] Meneses C Silva B Medeiros B Serrato R Johnston-Monje D. A metagenomic advance for the cloning and characterization of a cellulase from red rice crop residues. Molecules (2016) 21:E831. 10.3390/molecules21070831 27347917 PMC6274478

[29] Bakker PA Berendsen RL Doornbos RF Wintermans PC Pieterse CM. The rhizosphere revisited: root microbiomics. Front Plant Sci. (2013) 4:165. 10.3389/fpls.2013.00165 23755059 PMC3667247

[30] Sasse J Martinoia E Northen T. Feed your friends: do plant exudates shape the root microbiome? Trends Plant Sci. (2018) 23:25–41. 10.1016/j.tplants.2017.09.003 29050989

[31] Bai Y Muller DB Srinivas G Garrido-Oter R Potthoff E Rott M. Functional overlap of the Arabidopsis leaf and root microbiota. Nature (2015) 528:364–9. 10.1038/nature16192 26633631

[32] Bakker P Pieterse CMJ de Jonge R Berendsen RL. The soil-borne legacy. Cell (2018) 172:1178–80. 10.1016/j.cell.2018.02.024 29522740

[33] Haas D Defago G. Biological control of soil-borne pathogens by fluorescent pseudomonads. Nat Rev Microbiol. (2005) 3:307–19. 10.1038/nrmicro1129 15759041

[34] Pieterse CM Zamioudis C Berendsen RL Weller DM Van Wees SC Bakker PA. Induced systemic resistance by beneficial microbes. Annu Rev Phytopathol. (2014) 52:347–75. 10.1146/annurev-phyto-082712-102340 24906124

[35] Weller DM Raaijmakers JM Gardener BB Thomashow LS. Microbial populations responsible for specific soil suppressiveness to plant pathogens. Annu Rev Phytopathol. (2002) 40:309–48. 10.1146/annurev.phyto.40.030402.110010 12147763

[36] Mendes LW Raaijmakers JM de Hollander M Mendes R Tsai SM. Influence of resistance breeding in common bean on rhizosphere microbiome composition and function. ISME J. (2018) 12:212–24. 10.1038/ismej.2017.158 29028000 PMC5739014

[37] Pajarillo EA Chae JP Balolong MP Kim HB Seo KS Kang DK. Pyrosequencing-based analysis of fecal microbial communities in three purebred pig lines. J Microbiol. (2014) 52:646–51. 10.1007/s12275-014-4270-2 25047525

[38] Kim HB Isaacson RE. The pig gut microbial diversity: understanding the pig gut microbial ecology through the next generation high throughput sequencing. Vet Microbiol. (2015) 177:242–51. 10.1016/j.vetmic.2015.03.014 25843944

[39] Gresse R Chaucheyras-Durand F Fleury MA Van de Wiele T Forano E Blanquet-Diot S. Gut microbiota dysbiosis in postweaning piglets: understanding the keys to health. Trends Microbiol. (2017) 25:851–73. 10.1016/j.tim.2017.05.004 28602521

[40] Looft T Allen HK Cantarel BL Levine UY Bayles DO Alt DP. Bacteria, phages and pigs: the effects of in-feed antibiotics on the microbiome at different gut locations. ISME J. (2014) 8:1566–76. 10.1038/ismej.2014.12 24522263 PMC4817603

[41] Johnson TA Looft T Severin AJ Bayles DO Nasko DJ Wommack KE. The in-feed antibiotic carbadox induces phage gene transcription in the swine gut microbiome. mBio (2017) 8:e00709-17. 10.1128/mBio.00709-17 28790203 PMC5550749

[42] Brown DC Maxwell CV Erf GF Davis ME Singh S Johnson ZB. The influence of different management systems and age on intestinal morphology, immune cell numbers and mucin production from goblet cells in post-weaning pigs. Vet Immunol Immunopathol. (2006) 111:187–98. 10.1016/j.vetimm.2005.12.006 16621019

[43] Danielsson R Dicksved J Sun L Gonda H Muller B Schnurer A. Methane production in dairy cows correlates with rumen methanogenic and bacterial community structure. Front Microbiol. (2017) 8:226. 10.3389/fmicb.2017.00226 28261182 PMC5313486

[44] Uyeno Y Sekiguchi Y Kamagata Y. rRNA-based analysis to monitor succession of faecal bacterial communities in Holstein calves. Lett Appl Microbiol. (2010) 51:570–7. 10.1111/j.1472-765X.2010.02937.x 20849397

[45] Uyeno Y Sekiguchi Y Tajima K Takenaka A Kurihara M Kamagata Y. An rRNA-based analysis for evaluating the effect of heat stress on the rumen microbial composition of *Holstein heifers*. Anaerobe (2010) 16:27–33. 10.1016/j.anaerobe.2009.04.006 19446029

[46] Meale SJ Li S Azevedo P Derakhshani H Plaizier JC Khafipour E Development of ruminal and fecal microbiomes are affected by weaning but not weaning strategy in dairy calves. Front Microbiol. (2016) 7:582 10.3389/fmicb.2016.00582 27199916 PMC4853645

[47] Khafipour E Li S Plaizier JC Krause DO. Rumen microbiome composition determined using two nutritional models of subacute ruminal acidosis. Appl Environ Microbiol. (2009) 75:7115–24. 10.1128/AEM.00739-09 19783747 PMC2786511

[48] Kleen JL Hooijer GA Rehage J Noordhuizen JP. Subacute ruminal acidosis (SARA): a review. J Vet Med A Physiol Pathol Clin Med. (2003) 50:406–14. 10.1046/j.1439-0442.2003.00569.x 14633219

[49] Gozho GN Plaizier JC Krause DO Kennedy AD Wittenberg KM. Subacute ruminal acidosis induces ruminal lipopolysaccharide endotoxin release and triggers an inflammatory response. J Dairy Sci. (2005) 88:1399–403. 10.3168/jds.S0022-0302(05)72807-1 15778308

[50] Oakley BB Lillehoj HS Kogut MH Kim WK Maurer JJ Pedroso A. The chicken gastrointestinal microbiome. FEMS Microbiol Lett. (2014) 360:100–12. 10.1111/1574-6968.12608 25263745

[51] Rantala M Nurmi E. Prevention of the growth of *Salmonella infantis* in chicks by the flora of the alimentary tract of chickens. Br Poult Sci. (1973) 14:627–30. 10.1080/00071667308416073 4759990

[52] Lee KW Lillehoj HS Jeong W Jeoung HY An DJ. Avian necrotic enteritis: experimental models, host immunity, pathogenesis, risk factors, and vaccine development. Poult Sci. (2011) 90:1381–90. 10.3382/ps.2010-01319 21673152

[53] Antonissen G Eeckhaut V Van Driessche K Onrust L Haesebrouck F Ducatelle R. Microbial shifts associated with necrotic enteritis. Avian Pathol. (2016) 45:308–12. 10.1080/03079457.2016.1152625 26950294

[54] Van Immerseel F De Buck J Pasmans F Huyghebaert G Haesebrouck F Ducatelle R *Clostridium perfringens* in poultry: an emerging threat for animal and public health. Avian Pathol. (2004) 33:537–49. 10.1080/03079450400013162 15763720

[55] Collier CT Hofacre CL Payne AM Anderson DB Kaiser P Mackie RI. Coccidia-induced mucogenesis promotes the onset of necrotic enteritis by supporting *Clostridium perfringens* growth. Vet Immunol Immunopathol. (2008) 122:104–15. 10.1016/j.vetimm.2007.10.014 18068809

[56] Ahern PP Faith JJ Gordon JI. Mining the human gut microbiota for effector strains that shape the immune system. Immunity (2014) 40:815–23. 10.1016/j.immuni.2014.05.012 24950201 PMC4118768

[57] Geuking MB Koller Y Rupp S McCoy KD. The interplay between the gut microbiota and the immune system. Gut Microbes (2014) 5:411–8. 10.4161/gmic.29330 24922519 PMC4153781

[58] Gensollen T Iyer SS Kasper DL Blumberg RS. How colonization by microbiota in early life shapes the immune system. Science (2016) 352:539–44. 10.1126/science.aad9378 27126036 PMC5050524

[59] Gomez de Aguero M Ganal-Vonarburg SC Fuhrer T Rupp S Uchimura Y Li H The maternal microbiota drives early postnatal innate immune development. Science (2016) 351:1296–302. 10.1126/science.aad2571 26989247

[60] Lozupone CA Stombaugh JI Gordon JI Jansson JK Knight R. Diversity, stability and resilience of the human gut microbiota. Nature (2012) 489:220–30. 10.1038/nature11550 22972295 PMC3577372

[61] Passos M Moraes-Filho JP. Intestinal microbiota in digestive diseases. Arq Gastroenterol. (2017) 54:255–62. 10.1590/s0004-2803.201700000-31 28723981

[62] Collado MC Rautava S Isolauri E Salminen S. Gut microbiota: a source of novel tools to reduce the risk of human disease? Pediatr Res. (2015) 77:182–8. 10.1038/pr.2014.173 25335085

[63] Mahmoodpoor F Rahbar Saadat Y Barzegari A Ardalan M Zununi Vahed S. The impact of gut microbiota on kidney function and pathogenesis. Biomed. Pharmacother. (2017) 93:412–9. 10.1016/j.biopha.2017.06.066 28654798

[64] Belkaid Y Hand TW. Role of the microbiota in immunity and inflammation. Cell (2014) 157:121–41. 10.1016/j.cell.2014.03.011 24679531 PMC4056765

[65] Lee WJ Hase K. Gut microbiota-generated metabolites in animal health and disease. Nat Chem Biol. (2014) 10:416–24. 10.1038/nchembio.1535 24838170

[66] Momeni-Moghaddam P Keyvanshokooh S Ziaei-Nejad S Parviz Salati A Pasha-Zanoosi H. Effects of mannan oligosaccharide supplementation on growth, some immune responses and gut lactic acid bacteria of common carp (*Cyprinus carpio*) fingerlings. Vet Res Forum (2015) 6:239–44. 26893815 PMC4611979

[67] Torrecillas S Makol A Caballero MJ Montero D Dhanasiri AK Sweetman J. Effects on mortality and stress response in European sea bass, *Dicentrarchus labrax* (L.), fed mannan oligosaccharides (MOS) after *Vibrio anguillarum* exposure. J Fish Dis. (2012) 35:591–602. 10.1111/j.1365-2761.2012.01384.x 22690841

[68] Torrecillas S Makol A Caballero MJ Montero D Robaina L Real F. Immune stimulation and improved infection resistance in European sea bass (*Dicentrarchus labrax*) fed mannan oligosaccharides. Fish Shellfish Immunol. (2007) 23:969–81. 10.1016/j.fsi.2007.03.007 17766145

[69] Torrecillas S Makol A Benitez-Santana T Caballero MJ Montero D Sweetman J. Reduced gut bacterial translocation in European sea bass (*Dicentrarchus labrax*) fed mannan oligosaccharides (MOS). Fish Shellfish Immunol. (2011) 30:674–81. 10.1016/j.fsi.2010.12.020 21195771

[70] Burr G Hume M Ricke S Nisbet D Gatlin D III. *In vitro* and *in vivo* evaluation of the prebiotics GroBiotic-A, inulin, mannanoligosaccharide, and galactooligosaccharide on the digestive microbiota and performance of hybrid striped bass (*Morone chrysops* × *Morone saxatilis*). Microb Ecol. (2010) 59:187–98. 10.1007/s00248-009-9597-6 19844649

[71] Dimitroglou A Merrifield DL Moate R Davies SJ Spring P Sweetman J. Dietary mannan oligosaccharide supplementation modulates intestinal microbial ecology and improves gut morphology of rainbow trout, *Oncorhynchus mykiss* (Walbaum). J Anim Sci. (2009) 87:3226–34. 10.2527/jas.2008-1428 19617514

[72] Carbone D Faggio C. Importance of prebiotics in aquaculture as immunostimulants. Effects on immune system of *Sparus aurata* and *Dicentrarchus labrax*. Fish Shellfish Immunol. (2016) 54:172–8. 10.1016/j.fsi.2016.04.011 27074444

[73] Azimirad M Meshkini S Ahmadifard N Hoseinifar SH. The effects of feeding with synbiotic (*Pediococcus acidilactici* and fructooligosaccharide) enriched adult Artemia on skin mucus immune responses, stress resistance, intestinal microbiota and performance of angelfish (*Pterophyllum scalare*). Fish Shellfish Immunol. (2016) 54:516–22. 10.1016/j.fsi.2016.05.001 27150050

[74] Hoseinifar SH Soleimani N Ringo E. Effects of dietary fructo-oligosaccharide supplementation on the growth performance, haemato-immunological parameters, gut microbiota and stress resistance of common carp (*Cyprinus carpio*) fry. Br J Nutr. (2014) 112:1296–302. 10.1017/S0007114514002037 25313574

[75] Akrami R Iri Y Rostami HK Razeghi Mansour M. Effect of dietary supplementation of fructooligosaccharide (FOS) on growth performance, survival, lactobacillus bacterial population and hemato-immunological parameters of stellate sturgeon (*Acipenser stellatus*) juvenile. Fish Shellfish Immunol. (2013) 35:1235–9. 10.1016/j.fsi.2013.07.039 23973846

[76] Ran C Hu J Liu W Liu Z He S Dan BC. Thymol and carvacrol affect hybrid tilapia through the combination of direct stimulation and an intestinal microbiota-mediated effect: insights from a germ-free zebrafish model. J Nutr. (2016) 146:1132–40. 10.3945/jn.115.229377 27075912

[77] Banerjee G Ray AK. The advancement of probiotics research and its application in fish farming industries. Res Vet Sci. (2017) 115:66–77. 10.1016/j.rvsc.2017.01.016 28157611

[78] Makridis P Papandroulakis N Divanach P. Use of Phaeobacter sp. probiotic bacteria for the rearing of sea bass larvae (*Dicentrarchus labrax*). Commun Agric Appl Biol Sci. (2013) 78:259–61. 25141684

[79] Rasmussen BB Grotkjaer T D'Alvise PW Yin G Zhang F Bunk B. *Vibrio anguillarum* is genetically and phenotypically unaffected by long-term continuous exposure to the antibacterial compound tropodithietic acid. Appl Environ Microbiol. (2016) 82:4802–10. 10.1128/AEM.01047-16 27235441 PMC4984299

[80] Cerezuela R Meseguer J Esteban MA. Effects of dietary inulin, *Bacillus subtilis* and microalgae on intestinal gene expression in gilthead seabream (*Sparus aurata* L.). Fish Shellfish Immunol. (2013) 34:843–8. 10.1016/j.fsi.2012.12.026 23318995

[81] Larkin RP. Soil health paradigms and implications for disease management. Annu Rev Phytopathol. (2015) 53:199–221. 10.1146/annurev-phyto-080614-120357 26002292

[82] Ling N Zhu C Xue C Chen H Duan Y Peng C Insight into how organic amendments can shape the soil microbiome in long-term field experiments as revealed by network analysis. Soil Biol Biochem. (2016) 99:137–49. 10.1016/j.soilbio.2016.05.005

[83] Inderbitzin P Ward J Barbella A Solares N Izyumin D Burman P. Soil microbiomes associated with verticillium wilt-suppressive broccoli and chitin amendments are enriched with potential biocontrol agents. Phytopathology (2018) 108:31–43. 10.1094/PHYTO-07-17-0242-R 28876209

[84] Lehmann J Rillig MC Thies J Masiello CA Hockaday WC Crowley D Biochar effects on soil biota–a review. Soil Biol Biochem. (2011) 43:1812–36. 10.1016/j.soilbio.2011.04.022

[85] Jenkins JR Viger M Arnold EC Harris ZM Ventura M Miglietta F Biochar alters the soil microbiome and soil function: results of next-generation amplicon sequencing across Europe. GCB Bioenergy (2017) 9:591–612. 10.1111/gcbb.12371

[86] Elad Y David DR Harel YM Borenshtein M Kalifa HB Silber A. Induction of systemic resistance in plants by biochar, a soil-applied carbon sequestering agent. Phytopathology (2010) 100:913–21. 10.1094/PHYTO-100-9-0913 20701489

[87] Stringlis IA Yu K Feussner K De Jonge R Van Bentum S Van Verk MC. MYB72-dependent coumarin exudation shapes root microbiome assembly to promote plant health. Proc Natl Acad Sci USA (2018) 115:E5213–22. 10.1073/pnas.1722335115 29686086 PMC5984513

[88] Compant S Duffy B Nowak J Clement C Barka EA. Use of plant growth-promoting bacteria for biocontrol of plant diseases: principles, mechanisms of action, and future prospects. Appl Environ Microbiol. (2005) 71:4951–9. 10.1128/AEM.71.9.4951-4959.2005 16151072 PMC1214602

[89] Timmusk S Behers L Muthoni J Muraya A Aronsson AC. Perspectives and challenges of microbial application for crop improvement. Front Plant Sci. (2017) 8:49. 10.3389/fpls.2017.00049 28232839 PMC5299024

[90] Ongena M Jacques P Delfosse P Thonart P. Unusual traits of the pyoverdin-mediated iron acquisition system in Pseudomonas putida strain BTP1. Biometals (2002) 15:1–13. 10.1023/A:1013157824411 11860018

[91] Ongena M Jacques P Toure Y Destain J Jabrane A Thonart P. Involvement of fengycin-type lipopeptides in the multifaceted biocontrol potential of Bacillus subtilis. Appl Microbiol Biotechnol. (2005) 69:29–38. 10.1007/s00253-005-1940-3 15742166

[92] Ongena M Jacques P. Bacillus lipopeptides: versatile weapons for plant disease biocontrol. Trends Microbiol. (2008) 16:115–25. 10.1016/j.tim.2007.12.009 18289856

[93] Lugtenberg BJ Dekkers L Bloemberg GV. Molecular determinants of rhizosphere colonization by Pseudomonas. Annu Rev Phytopathol. (2001) 39:461–90. 10.1146/annurev.phyto.39.1.461 11701873

[94] Fravel DR. Commercialization and implementation of biocontrol. Annu Rev Phytopathol. (2005) 43:337–59. 10.1146/annurev.phyto.43.032904.092924 16078888

[95] Chapelle E Mendes R Bakker PA Raaijmakers JM. Fungal invasion of the rhizosphere microbiome. ISME J. (2016) 10:265–8. 10.1038/ismej.2015.82 26023875 PMC4681858

[96] Berendsen RL Vismans G Yu K Song Y de Jonge R Burgman WP. Disease-induced assemblage of a plant-beneficial bacterial consortium. ISME J. (2018) 12:1496–507. 10.1038/s41396-018-0093-1 29520025 PMC5956071

[97] Perez-Jaramillo JE Mendes R Raaijmakers JM. Impact of plant domestication on rhizosphere microbiome assembly and functions. Plant Mol Biol. (2016) 90:635–44. 10.1007/s11103-015-0337-7 26085172 PMC4819786

[98] Jiao LF Ke YL Xiao K Song ZH Hu CH Shi B. Effects of cello-oligosaccharide on intestinal microbiota and epithelial barrier function of weanling pigs. J Anim Sci. (2015) 93:1157–64. 10.2527/jas.2014-8248 26020893

[99] Alizadeh A Akbari P Difilippo E Schols HA Ulfman LH Schoterman MH. The piglet as a model for studying dietary components in infant diets: effects of galacto-oligosaccharides on intestinal functions. Br J Nutr. (2016) 115:605–18. 10.1017/S0007114515004997 26653138

[100] Hoeflinger JL Kashtanov DO Cox SB Dowd SE Jouni ZE Donovan SM. Characterization of the intestinal Lactobacilli community following galactooligosaccharides and polydextrose supplementation in the neonatal piglet. PLoS ONE (2015) 10:e0135494. 10.1371/journal.pone.0135494 26275147 PMC4537252

[101] Le Bourgot C Le Normand L Formal M Respondek F Blat S Apper E Maternal short-chain fructo-oligosaccharide supplementation increases intestinal cytokine secretion, goblet cell number, butyrate concentration and *Lawsonia intracellularis* humoral vaccine response in weaned pigs. Br J Nutr. (2017) 117:83–92. 10.1017/S0007114516004268 28115029

[102] Trevisi P De Filippi S Minieri L Mazzoni M Modesto M Biavati B. Effect of fructo-oligosaccharides and different doses of *Bifidobacterium animalis* in a weaning diet on bacterial translocation and Toll-like receptor gene expression in pigs. Nutrition (2008) 24:1023–9. 10.1016/j.nut.2008.04.008 18562167

[103] Barba-Vidal E Martin-Orue SM Castillejos L. Review: are we using probiotics correctly in post-weaning piglets? Animal (2018). [Epub ahead of print]. 10.1017/S1751731118000873 29720287

[104] Ghosh S Mehla RK. Influence of dietary supplementation of prebiotics (mannanoligosaccharide) on the performance of crossbred calves. Trop Anim Health Prod. (2012) 44:617–22. 10.1007/s11250-011-9944-8 21805307

[105] Castro JJ Gomez A White BA Mangian HJ Loften JR Drackley JK Changes in the intestinal bacterial community, short-chain fatty acid profile, and intestinal development of preweaned Holstein calves. 1. Effects of prebiotic supplementation depend on site and age. J Dairy Sci. (2016) 99:9682–702. 10.3168/jds.2016-11006 27720150

[106] Grand E Respondek F Martineau C Detilleux J Bertrand G. Effects of short-chain fructooligosaccharides on growth performance of preruminant veal calves. J Dairy Sci. (2013) 96:1094–101. 10.3168/jds.2011-4949 23200477

[107] Fleige S Preissinger W Meyer HH Pfaffl MW. The immunomodulatory effect of lactulose on *Enterococcus faecium* fed preruminant calves. J Anim Sci. (2009) 87:1731–8. 10.2527/jas.2007-0494 19098237

[108] Duncan SH Louis P Flint HJ. Lactate-utilizing bacteria, isolated from human feces, that produce butyrate as a major fermentation product. Appl Environ Microbiol. (2004) 70:5810–7. 10.1128/AEM.70.10.5810-5817.2004 15466518 PMC522113

[109] Louis P Flint HJ. Diversity, metabolism and microbial ecology of butyrate-producing bacteria from the human large intestine. FEMS Microbiol Lett. (2009) 294:1–8. 10.1111/j.1574-6968.2009.01514.x 19222573

[110] Ghorbani GR Morgavi DP Beauchemin KA Leedle JA. Effects of bacterial direct-fed microbials on ruminal fermentation, blood variables, and the microbial populations of feedlot cattle. J Anim Sci. (2002) 80:1977–85. 10.2527/2002.8071977x 12162668

[111] Nocek JE Kautz WP Leedle JA Allman JG. Ruminal supplementation of direct-fed microbials on diurnal pH variation and *in situ* digestion in dairy cattle. J Dairy Sci. (2002) 85:429–33. 10.3168/jds.S0022-0302(02)74091-5 11915864

[112] Klieve AV Hennessy D Ouwerkerk D Forster RJ Mackie RI Attwood GT. Establishing populations of *Megasphaera elsdenii* YE 34 and *Butyrivibrio fibrisolvens* YE 44 in the rumen of cattle fed high grain diets. J Appl Microbiol. (2003) 95:621–30. 10.1046/j.1365-2672.2003.02024.x 12911711

[113] Nocek JE Socha MT Tomlinson DJ. The effect of trace mineral fortification level and source on performance of dairy cattle. J Dairy Sci. (2006) 89:2679–93. 10.3168/jds.S0022-0302(06)72344-X 16772587

[114] Uyeno Y Shigemori S Shimosato T. Effect of probiotics/prebiotics on cattle health and productivity. Microb Environ. (2015) 30:126–32. 10.1264/jsme2.ME14176 26004794 PMC4462921

[115] Pourabedin M. Zhao X. (2015). Prebiotics and gut microbiota in chickens. FEMS Microbiol Lett. 362:fnv122 10.1093/femsle/fnv122 26208530

[116] Vael C Desager K. The importance of the development of the intestinal microbiota in infancy. Curr Opin Pediatr. (2009) 21:794–800. 10.1097/MOP.0b013e328332351b 19770768

[117] Donovan SM Comstock SS. Human milk oligosaccharides influence neonatal mucosal and systemic immunity. Ann Nutr Metab. (2016) 69(Suppl 2):42–51. 10.1159/000452818 28103609 PMC6392703

[118] Arslanoglu S Moro GE Schmitt J Tandoi L Rizzardi S Boehm G. Early dietary intervention with a mixture of prebiotic oligosaccharides reduces the incidence of allergic manifestations and infections during the first two years of life. J Nutr. (2008) 138:1091–5. 10.1093/jn/138.6.1091 18492839

[119] Gourbeyre P Desbuards N Gremy G Tranquet O Champ M Denery-Papini S. Perinatal and postweaning exposure to galactooligosaccharides/inulin prebiotics induced biomarkers linked to tolerance mechanism in a mouse model of strong allergic sensitization. J Agric Food Chem. (2013) 61:6311–20. 10.1021/jf305315g 23746232

[120] Verheijden KA Braber S Leusink-Muis T Thijssen S Boon L Kraneveld AD Regulatory T cell depletion abolishes the protective effect of dietary galacto-oligosaccharides on eosinophilic airway inflammation in house dust mite-induced asthma in mice. J Nutr. (2016). 146, 831–837. 10.3945/jn.115.224402 26962188

[121] Vos AP Knol J Stahl B M'Rabet L Garssen J. Specific prebiotic oligosaccharides modulate the early phase of a murine vaccination response. Int. Immunopharmacol. (2010) 10:619–25. 10.1016/j.intimp.2010.02.014 20206301

[122] Arslanoglu S Moro GE Boehm G. Early supplementation of prebiotic oligosaccharides protects formula-fed infants against infections during the first 6 months of life. J Nutr. (2007) 137:2420–4. 10.1093/jn/137.11.2420 17951479

[123] Kukkonen K Savilahti E Haahtela T Juntunen-Backman K Korpela R Poussa T. Long-term safety and impact on infection rates of postnatal probiotic and prebiotic (synbiotic) treatment: randomized, double-blind, placebo-controlled trial. Pediatrics (2008) 122:8–12. 10.1542/peds.2007-1192 18595980

[124] Silk DB Davis A Vulevic J Tzortzis G Gibson GR. Clinical trial: the effects of a trans-galactooligosaccharide prebiotic on faecal microbiota and symptoms in irritable bowel syndrome. Aliment Pharmacol Ther. (2009) 29:508–18. 10.1111/j.1365-2036.2008.03911.x 19053980

[125] Drakoularakou A Tzortzis G Rastall RA Gibson GR. A double-blind, placebo-controlled, randomized human study assessing the capacity of a novel galacto-oligosaccharide mixture in reducing travellers' diarrhoea. Eur J Clin Nutr. (2010) 64:146–52. 10.1038/ejcn.2009.120 19756029

[126] Parnell JA Reimer RA. Weight loss during oligofructose supplementation is associated with decreased ghrelin and increased peptide YY in overweight and obese adults. Am J Clin Nutr. (2009) 89:1751–9. 10.3945/ajcn.2009.27465 19386741 PMC3827013

[127] Qamar TR Syed F Nasir M Rehman H Zahid MN Liu RH. Novel combination of prebiotics galacto-oligosaccharides and inulin-inhibited aberrant crypt foci formation and biomarkers of colon cancer in wistar rats. Nutrients (2016) 8:E465. 10.3390/nu8080465 27490566 PMC4997378

[128] Qamar TR Iqbal S Syed F Nasir M Rehman H Iqbal MA. Impact of novel prebiotic galacto-oligosaccharides on various biomarkers of colorectal cancer in wister rats. Int J Mol Sci. (2017) 18:E1785. 10.3390/ijms18091785 28858205 PMC5618473

[129] Abrams SA Griffin IJ Hawthorne KM Liang L Gunn SK Darlington G. A combination of prebiotic short- and long-chain inulin-type fructans enhances calcium absorption and bone mineralization in young adolescents. Am J Clin Nutr. (2005) 82:471–6. 10.1093/ajcn/82.2.471 16087995

[130] Slavin J. Fiber and prebiotics: mechanisms and health benefits. Nutrients (2013) 5:1417–35. 10.3390/nu5041417 23609775 PMC3705355

[131] Bouhnik Y Raskine L Simoneau G Paineau D Bornet F. The capacity of short-chain fructo-oligosaccharides to stimulate faecal bifidobacteria: a dose-response relationship study in healthy humans. Nutr J. (2006) 5:8. 10.1186/1475-2891-5-8 16569219 PMC1448190

[132] Brunser O Gotteland M Cruchet S Figueroa G Garrido D Steenhout P. Effect of a milk formula with prebiotics on the intestinal microbiota of infants after an antibiotic treatment. Pediatr Res. (2006) 59:451–6. 10.1203/01.pdr.0000198773.40937.61 16492988

[133] Scholtens PA Alliet P Raes M Alles MS Kroes H Boehm G. Fecal secretory immunoglobulin A is increased in healthy infants who receive a formula with short-chain galacto-oligosaccharides and long-chain fructo-oligosaccharides. J Nutr. (2008) 138:1141–7. 10.1093/jn/138.6.1141 18492847

[134] Scholtens S Wijga AH Smit HA Brunekreef B de Jongste JC Gerritsen J. Long-chain polyunsaturated fatty acids in breast milk and early weight gain in breast-fed infants. Br J Nutr. (2009) 101:116–21. 10.1017/S0007114508993521 18492299

[135] Meijer K de Vos P Priebe MG. Butyrate and other short-chain fatty acids as modulators of immunity: what relevance for health? Curr Opin Clin Nutr Metab Care (2010) 13:715–21. 10.1097/MCO.0b013e32833eebe5 20823773

[136] McLoughlin RF Berthon BS Jensen ME Baines KJ Wood LG. Short-chain fatty acids, prebiotics, synbiotics, and systemic inflammation: a systematic review and meta-analysis. Am J Clin Nutr. (2017) 106:930–45. 10.3945/ajcn.117.156265 28793992

[137] Postler TS Ghosh S. Understanding the holobiont: how microbial metabolites affect human health and shape the immune system. Cell Metab. (2017) 26:110–30. 10.1016/j.cmet.2017.05.008 28625867 PMC5535818

[138] Shoaf K Mulvey GL Armstrong GD Hutkins RW. Prebiotic galactooligosaccharides reduce adherence of enteropathogenic *Escherichia coli* to tissue culture cells. Infect Immun. (2006) 74:6920–8. 10.1128/IAI.01030-06 16982832 PMC1698067

[139] Jantscher-Krenn E Bode L. Human milk oligosaccharides and their potential benefits for the breast-fed neonate. Minerva Pediatr. (2012) 64:83–99. 22350049

[140] Douellou T Montel MC Thevenot Sergentet D. Invited review: anti-adhesive properties of bovine oligosaccharides and bovine milk fat globule membrane-associated glycoconjugates against bacterial food enteropathogens. J Dairy Sci. (2017) 100:3348–59. 10.3168/jds.2016-11611 28161162

[141] Shokryazdan P Faseleh Jahromi M Navidshad B Liang JB. Effects of prebiotics on immune system and cytokine expression. Med Microbiol Immunol. (2017) 206:1–9. 10.1007/s00430-016-0481-y 27704207

[142] Wilson B Whelan K. Prebiotic inulin-type fructans and galacto-oligosaccharides: definition, specificity, function, and application in gastrointestinal disorders. J Gastroenterol. Hepat. (2017) 32(Suppl 1):64–8. 10.1111/jgh.13700 28244671

[143] Zenhom M Hyder A de Vrese M Heller KJ Roeder T Schrezenmeir J. Prebiotic oligosaccharides reduce proinflammatory cytokines in intestinal Caco-2 cells via activation of PPARgamma and peptidoglycan recognition protein 3. J Nutr. (2011) 141:971–7. 10.3945/jn.110.136176 21451128

[144] Bode L. The functional biology of human milk oligosaccharides. Early Hum Dev. (2015) 91:619–22. 10.1016/j.earlhumdev.2015.09.001 26375354

[145] Perdijk O van Splunter M Savelkoul HFJ Brugman S van Neerven RJJ. Cow's milk and immune function in the respiratory tract: potential mechanisms. Front Immunol. (2018) 9:143. 10.3389/fimmu.2018.00143 29483908 PMC5816034

[146] Perdijk O van Neerven RJJ Meijer B Savelkoul HFJ Brugman S. Induction of human tolerogenic dendritic cells by 3'-sialyllactose via TLR4 is explained by LPS contamination. Glycobiology (2018) 28:126–30. 10.1093/glycob/cwx106 29281012 PMC5993091

[147] Zhong Y Cai D Cai W Geng S Chen L Han T. Protective effect of galactooligosaccharide-supplemented enteral nutrition on intestinal barrier function in rats with severe acute pancreatitis. Clin Nutr. (2009) 28:575–80. 10.1016/j.clnu.2009.04.026 19525042

[148] Searle LE Cooley WA Jones G Nunez A Crudgington B Weyer U. Purified galactooligosaccharide, derived from a mixture produced by the enzymic activity of *Bifidobacterium bifidum*, reduces *Salmonella enterica* serovar Typhimurium adhesion and invasion *in vitro* and *in vivo*. J Med Microbiol. (2010) 59:1428–39. 10.1099/jmm.0.022780-0 20798214

[149] Bhatia S Prabhu PN Benefiel AC Miller MJ Chow J Davis SR. Galacto-oligosaccharides may directly enhance intestinal barrier function through the modulation of goblet cells. Mol Nutr Food Res. (2015) 59:566–73. 10.1002/mnfr.201400639 25421108

[150] Akbari P Fink-Gremmels J Willems R Difilippo E Schols HA Schoterman MHC. Characterizing microbiota-independent effects of oligosaccharides on intestinal epithelial cells: insight into the role of structure and size: structure-activity relationships of non-digestible oligosaccharides. Eur J Nutr. (2017) 56:1919–30. 10.1007/s00394-016-1234-9 27295033 PMC5534205

[151] Garcia-Rodenas CL Bergonzelli GE Nutten S Schumann A Cherbut C Turini M. Nutritional approach to restore impaired intestinal barrier function and growth after neonatal stress in rats. J Pediatr Gastroenterol Nutr. (2006) 43:16–24. 10.1097/01.mpg.0000226376.95623.9f 16819372

[152] Cani PD Lecourt E Dewulf EM Sohet FM Pachikian BD Naslain D. Gut microbiota fermentation of prebiotics increases satietogenic and incretin gut peptide production with consequences for appetite sensation and glucose response after a meal. Am J Clin Nutr. (2009) 90:1236–43. 10.3945/ajcn.2009.28095 19776140

[153] Kaliannan K Wang B Li XY Kim KJ Kang JX. A host-microbiome interaction mediates the opposing effects of omega-6 and omega-3 fatty acids on metabolic endotoxemia. Sci Rep. (2015) 5:11276. 10.1038/srep11276 26062993 PMC4650612

[154] Pusceddu MM El Aidy S Crispie F O'Sullivan O Cotter P Stanton C. Correction: N-3 Polyunsaturated Fatty Acids (PUFAs) reverse the impact of early-life stress on the gut microbiota. PLoS ONE (2015) 10:e0142228. 10.1371/journal.pone.0142228 26517367 PMC4627768

[155] Pusceddu MM El Aidy S Crispie F O'Sullivan O Cotter P Stanton C N-3 Polyunsaturated Fatty Acids (PUFAs) reverse the impact of early-life stress on the gut microbiota. PLoS ONE (2015) 10:e0139721. 10.1371/journal.pone.0139721 PMC4591340 26426902

[156] Etxeberria U Fernandez-Quintela A Milagro FI Aguirre L Martinez JA Portillo MP. Impact of polyphenols and polyphenol-rich dietary sources on gut microbiota composition. J Agric Food Chem. (2013) 61:9517–33. 10.1021/jf402506c 24033291

[157] Ozdal T Sela DA Xiao J Boyacioglu D Chen F Capanoglu E. The reciprocal interactions between polyphenols and gut microbiota and effects on bioaccessibility. Nutrients (2016) 8:78. 10.3390/nu8020078 26861391 PMC4772042

[158] Tomas-Barberan FA Selma MV Espin JC. Interactions of gut microbiota with dietary polyphenols and consequences to human health. Curr Opin Clin Nutr Metab Care (2016) 19:471–6. 10.1097/MCO.0000000000000314 27490306

[159] Roca-Saavedra P Mendez-Vilabrille V Miranda JM Nebot C Cardelle-Cobas A Franco CM. Food additives, contaminants and other minor components: effects on human gut microbiota-a review. J Physiol Biochem. (2018) 74:69–83. 10.1007/s13105-017-0564-2 28488210

[160] Gerritsen J Smidt H Rijkers GT de Vos WM. Intestinal microbiota in human health and disease: the impact of probiotics. Genes Nutr. (2011) 6:209–40. 10.1007/s12263-011-0229-7 21617937 PMC3145058

[161] Ismail IH Licciardi PV Tang ML. Probiotic effects in allergic disease. J Paediatr Child Health (2013) 49:709–15. 10.1111/jpc.12175 23574636

[162] Power SE O'Toole PW Stanton C Ross RP Fitzgerald GF. Intestinal microbiota, diet and health. Br J Nutr. (2014) 111:387–402. 10.1017/S0007114513002560 23931069

[163] Sanchez B Delgado S Blanco-Miguez A Lourenco A Gueimonde M Margolles A. Probiotics, gut microbiota, and their influence on host health and disease. Mol. Nutr. Food Res. (2017) 61:1600240. 10.1002/mnfr.201600240 27500859

[164] Liu S Hu P Du X Zhou T Pei X. *Lactobacillus rhamnosus* GG supplementation for preventing respiratory infections in children: a meta-analysis of randomized, placebo-controlled trials. Indian Pediatr. (2013) 50:377–81. 10.1007/s13312-013-0123-z 23665598

[165] Inoue Y Shimojo N. Microbiome/microbiota and allergies. Semin Immunopathol. (2015) 37:57–64. 10.1007/s00281-014-0453-5 25326106

[166] Carabotti M Scirocco A Maselli MA Severi C. The gut-brain axis: interactions between enteric microbiota, central and enteric nervous systems. Ann Gastroenterol. (2015) 28:203–9. 25830558 PMC4367209

[167] Umbrello G Esposito S. Microbiota and neurologic diseases: potential effects of probiotics. J Transl Med. (2016) 14:298. 10.1186/s12967-016-1058-7 27756430 PMC5069982

[168] Goulet O. Potential role of the intestinal microbiota in programming health and disease. Nutr Rev. (2015) 73(Suppl 1):32–40. 10.1093/nutrit/nuv039 26175488

[169] Morrow LE Gogineni V Malesker MA. Probiotic, prebiotic, and synbiotic use in critically ill patients. Curr Opin Crit Care (2012) 18:186–91. 10.1097/MCC.0b013e3283514b17 22343306

[170] Bron PA Kleerebezem M Brummer RJ Cani PD Mercenier A MacDonald TT. Can probiotics modulate human disease by impacting intestinal barrier function? Br J Nutr. (2017) 117:93–107. 10.1017/S0007114516004037 28102115 PMC5297585

[171] Fong FL Shah NP Kirjavainen P El-Nezami H. Mechanism of action of probiotic bacteria on intestinal and systemic immunities and antigen-presenting cells. Int Rev Immunol. (2016) 35:179–88. 10.3109/08830185.2015.1096937 26606641

[172] Raaijmakers JM Bonsall RF Weller DM. Effect of population density of *Pseudomonas fluorescens* on production of 2,4-diacetylphloroglucinol in the rhizosphere of wheat. Phytopathology (1999) 89:470–5. 10.1094/PHYTO.1999.89.6.470 18944718

[173] Cha JY Han S Hong HJ Cho H Kim D Kwon Y. Microbial and biochemical basis of a Fusarium wilt-suppressive soil. ISME J. (2016) 10:119–29. 10.1038/ismej.2015.95 26057845 PMC4681868

[174] DePeters EJ George LW. Rumen transfaunation. Immunol Lett. (2014) 162:69–76. 10.1016/j.imlet.2014.05.009 25262872

[175] Weimer PJ Stevenson DM Mantovani HC Man SL. Host specificity of the ruminal bacterial community in the dairy cow following near-total exchange of ruminal contents. J Dairy Sci. (2010) 93:5902–12. 10.3168/jds.2010-3500 21094763

[176] Siegerstetter SC Petri RM Magowan E Lawlor PG Zebeli Q O'Connell NE. Fecal microbiota transplant from highly feed-efficient donors shows little effect on age-related changes in feed-efficiency-associated fecal microbiota from chickens. Appl. Environ. Microbiol. 84:e02330–17. 10.1128/AEM.02330-17 29101192 PMC5752867

[177] Hu L Geng S Li Y Cheng S Fu X Yue X. Exogenous fecal microbiota transplantation from local adult pigs to crossbred newborn piglets. Front Microbiol. (2017) 8:2663. 10.3389/fmicb.2017.02663 29375527 PMC5767267

[178] McCormack UM Curiao T Buzoianu SG Prieto ML Ryan T Varley P. Exploring a possible link between the intestinal microbiota and feed efficiency in pigs. Appl Environ Microbiol. (2017) 83:e00380-17. 10.1128/AEM.00380-17 28526795 PMC5514681

[179] Ribeiro GO Oss DB He Z Gruninger RJ Elekwachi C Forster RJ. Repeated inoculation of cattle rumen with bison rumen contents alters the rumen microbiome and improves nitrogen digestibility in cattle. Sci Rep. (2017) 7:1276. 10.1038/s41598-017-01269-3 28455495 PMC5430699

[180] Diao H Yan HL Xiao Y Yu B Zheng P He J Modulation of intestine development by fecal microbiota transplantation in suckling pigs. RSC Adv. (2018) 8:8709–20. 10.1039/C7RA11234C PMC9078615 35539874

[181] Donaldson EE Stanley D Hughes RJ Moore RJ. The time-course of broiler intestinal microbiota development after administration of cecal contents to incubating eggs. PeerJ (2017) 5:e3587. 10.7717/peerj.3587 28740754 PMC5522604

[182] Li SS Zhu A Benes V Costea PI Hercog R Hildebrand F. Durable coexistence of donor and recipient strains after fecal microbiota transplantation. Science (2016) 352:586–9. 10.1126/science.aad8852 27126044

[183] Kelly CR Kahn S Kashyap P Laine L Rubin D Atreja A. Update on fecal microbiota transplantation 2015: indications, methodologies, mechanisms, and outlook. Gastroenterology (2015) 149:223–37. 10.1053/j.gastro.2015.05.008 25982290 PMC4755303

[184] van Nood E Speelman P Nieuwdorp M Keller J Fecal microbiota transplantation: facts and controversies. Curr Opin Gastroenterol. (2014) 30:34–9. 10.1097/MOG.0000000000000024 24241245

[185] Smits LP Bouter KE de Vos WM Borody TJ Nieuwdorp M. Therapeutic potential of fecal microbiota transplantation. Gastroenterology (2013) 145:946–53. 10.1053/j.gastro.2013.08.058 24018052

[186] Chehoud C Dryga A Hwang Y Nagy-Szakal D Hollister EB Luna RA. Transfer of viral communities between human individuals during fecal microbiota transplantation. mBio (2016) 7:e00322. 10.1128/mBio.00322-16 27025251 PMC4817255

[187] Shen Z Zhu C Quan Y Yuan W Wu S Yang Z. Update on intestinal microbiota in Crohn's disease 2017: mechanisms, clinical application, adverse reactions, and outlook. J Gastroenterol Hepatol. (2017) 32:1804–12. 10.1111/jgh.13861 28677158

[188] Xu MQ Cao HL Wang WQ Wang S Cao XC Yan F. Fecal microbiota transplantation broadening its application beyond intestinal disorders. World J Gastroenterol. (2015) 21:102–11. 10.3748/wjg.v21.i1.102 25574083 PMC4284325

[189] Colman RJ Rubin DT. Fecal microbiota transplantation as therapy for inflammatory bowel disease: a systematic review and meta-analysis. J Crohns Colitis (2014) 8:1569–81. 10.1016/j.crohns.2014.08.006 25223604 PMC4296742

[190] Jorgensen SMD Hansen MM Erikstrup C Dahlerup JF Hvas CL. Faecal microbiota transplantation: establishment of a clinical application framework. Eur J Gastroenterol Hepatol. (2017) 29:e36–e45. 10.1097/MEG.0000000000000958 28863010

[191] Legrand T Catalano SR Wos-Oxley ML Stephens F Landos M Bansemer MS. The inner workings of the outer surface: skin and gill microbiota as indicators of changing gut health in yellowtail kingfish. Front Microbiol. (2017) 8:2664. 10.3389/fmicb.2017.02664 29379473 PMC5775239

[192] Brugman S Nieuwenhuis EE. Mucosal control of the intestinal microbial community. J Mol Med. (2010) 88:881–8. 10.1007/s00109-010-0639-9 20523962

[193] Litvak Y Byndloss MX Tsolis RM Baumler AJ. Dysbiotic Proteobacteria expansion: a microbial signature of epithelial dysfunction. Curr Opin Microbiol. (2017) 39:1–6. 10.1016/j.mib.2017.07.003 28783509

[194] Manfredo Vieira S Hiltensperger M Kumar V Zegarra-Ruiz D Dehner C Khan N Translocation of a gut pathobiont drives autoimmunity in mice and humans. Science (2018) 359:1156–61. 10.1126/science.aar7201 29590047 PMC5959731

